# A prospective study evaluating clopidogrel resistance among patients with ischemic cerebrovascular events across different ethnic groups in Israel

**DOI:** 10.3389/fstro.2026.1908099

**Published:** 2026-09-28

**Authors:** Fadi Shbat, Karine Beiruti Wiegler, Najib Dally, Elias Saad, Saher Srour, Elizabeth Eshel, Adi Sharabi Nov, Radi Shahien, Ronen Leker, Naaem Simaan

**Affiliations:** 1 Department of Neurology, Ziv Medical Center, Safed, Israel; 2 Azrieli Faculty of Medicine, Bar-Ilan University, Safed, Israel; 3 Research Wing, Ziv Medical Center, Safed, Israel; 4 Department of Internal Medicine B, Galilee Medical Centre, Nahariyah, Israel; 5 Department of Hematology, Ziv Medical Center, Safed, Israel; 6 Department of Radiology, Ziv Medical Center, Safed, Israel; 7 Hematology Laboratory, Ziv Medical Center, Safed, Israel; 8 Tel-Hai University of Kiryat Shmona and the Galilee, Tel Hai, Israel; 9 Department of Neurology, Hadassah-Hebrew University Medical Center, Jerusalem, Israel

**Keywords:** antiplatelet therapy, clopidogrel resistance, high on-treatment platelet reactivity, ischemic stroke, secondary stroke prevention, VASP phosphorylation assay

## Abstract

**Introduction:**

Clopidogrel is commonly used for secondary prevention after ischemic cerebrovascular events, but substantial interindividual variability in pharmacodynamics response has been reported. Individuals demonstrating resistance to clopidogrel face a higher likelihood of recurrent ischemic events. This study evaluated clopidogrel responsiveness among Jewish and Arab patients treated within the same regional healthcare setting.

**Methods:**

This prospective cohort included 102 patients with ischemic cerebrovascular events (acute ischemic stroke or transient ischemic attack) receiving clopidogrel for secondary prevention. Patients received either a loading dose (300 in 62 patients, 600 mg in 1 patient) followed by maintenance therapy or at least 7 days of 75 mg daily maintenance dosing (*n* = 39) prior to blood sampling. Platelet responsiveness was measured using the vasodilator-stimulated phosphoprotein (VASP) phosphorylation assay (PLT VASP/P2Y12, BioCytex, France). Patients were categorized according to VASP platelet reactivity index (PRI) as adequate responders (PRI <50%), intermediate responders (PRI 50%−69%), or patients with high residual platelet reactivity, operationally defined in this study as PRI ≥70%.

**Results:**

The study included 102 enrolled patients, of whom 57 (55.9%) were Jewish and 45 (44.1%) were Arab. A valid VASP-PRI measurement was available for 101 patients (57 Jewish and 44 Arab); one Arab patient had no evaluable PRI measurement. Therefore, analyses of platelet reactivity were based on 101 patients The median PRI for all patients was 65.5 (49.1–80.3), with no statistically significant differences between Jewish and Arab (*p* = 0.251). Overall, 42.6% were classified as non-responders, 29.7% as partial responders, and only 27.7% demonstrated adequate platelet inhibition. Jewish patients were significantly older than Arab patients (*p* = 0.008), whereas Arab patients had a significantly higher BMI (*p* = 0.019). Other demographic and clinical features were similar across groups.

**Conclusions:**

A high proportion of patients demonstrated elevated residual platelet reactivity according to the operational PRI threshold used in this study. No statistically significant ethnic difference in PRI was detected; however, the ethnic comparison was exploratory and the study was not powered to exclude modest between-group differences. Larger prospective studies incorporating CYP2C19 genotyping and longitudinal clinical outcomes are warranted.

## 1 Introduction

Clopidogrel, a P2Y12 receptor inhibitor, is a cornerstone of secondary prevention following ischemic cerebrovascular event (Powers et al., 2018). However, recurrent ischemic events remain common, despite guideline-directed therapy, partly due to interindividual variability in platelet inhibition during clopidogrel treatment (Hvas and Grove, 2017). Patients with high on-treatment platelet reactivity (HPR) may have an increased risk of recurrent vascular events, although the clinical implications of platelet-function measurements vary according to the assay, threshold, and clinical population (Zhou et al., 2022).

A universally accepted definition of clopidogrel resistance remains elusive (Leunissen et al., 2016). The terms resistance, hyporesponsiveness, high on-treatment platelet reactivity (HPR), and poor metabolizer are often used interchangeably, though they reflect distinct concepts. HPR is a pharmacodynamic measure indicating persistently elevated platelet reactivity despite treatment, whereas poor metabolizer status is a pharmacogenetic classification, most often related to CYP2C19 loss-of-function alleles (Caudle et al., 2017; Lee et al., 2022). Because VASP-PRI thresholds have been developed largely in coronary populations and no universally accepted stroke-specific threshold exists, terminology and cutoffs should be interpreted cautiously in ischemic cerebrovascular populations.

The prevalence of clopidogrel HPR varies widely (15.9%−49.5%), depending on assay methodology and cutoff values (Buonamici et al., 2007). Genetic polymorphisms affecting clopidogrel metabolism, drug-drug interactions, and comorbidities such as advanced age, diabetes, obesity, inflammation, and renal dysfunction, and poor treatment adherence, may contribute to this variability (Brandt et al., 2007; Buonamici et al., 2007; Geisler et al., 2008; Hochholzer et al., 2010, 2011; Hulot et al., 2006; Matetzky et al., 2004; Mega et al., 2009; Shuldiner et al., 2009; Sibbing et al., 2009; Trenk et al., 2008). Ethnic differences in CYP2C19 allele frequencies are well documented, with higher loss-of-function allele prevalence in East Asian populations compared with Caucasian populations (Ionova et al., 2020; Jeong et al., 2011).

Several studies have documented clopidogrel responsiveness in Jewish and Arab populations separately. Pharmacogenomic studies in Jewish populations (Scott et al., 2012; Yang et al., 2014) and observational studies in Arab cohorts (Ali and Elewa, 2019; Ali et al., 2022; Alkattan et al., 2022) have reported rates of clopidogrel resistance within globally observed ranges. However, these studies were conducted in distinct populations and healthcare settings. To our knowledge, no prior study has directly compared clopidogrel responsiveness between Arab and Jewish patients with ischemic cerebrovascular events within the same geographic and clinical environment.

Northern Israel (Galilee) provides a unique setting in which Jewish and Arab populations receive care within the same healthcare system, allowing exploration of ethnic differences while minimizing system-level confounders. The primary objective was to compare clopidogrel responsiveness between Arab and Jewish patients with ischemic cerebrovascular events in Northern Israel. The secondary objective was to describe the prevalence of elevated residual platelet reactivity according to the operational PRI threshold used in this study. Because prior pharmacogenomic and observational studies conducted separately in Jewish and Arab populations have generally reported overlapping ranges of clopidogrel responsiveness (Ali and Elewa, 2019; Ali et al., 2022; Alkattan et al., 2022; Scott et al., 2012; Yang et al., 2014), and because a cohort of this size is not powered to detect small between-group differences, the ethnic comparison in this study was designed and should be interpreted as an exploratory, hypothesis-generating analysis rather than a definitive test of ethnic differences in clopidogrel response.

## 2 Materiel and methods

### 2.1 Study population and design

This prospective study was conducted at the Neurology Department of Ziv Medical Center, Safed, and included 102 patients from different ethnic backgrounds presenting with ischemic cerebrovascular events (acute ischemic stroke or transient ischemic attack) and treated with clopidrogel for secondary prevention between November 2020 and November 2022 (Figure 1). This study was performed in accordance with the principles of the Declaration of Helsinki and received approval from the local Ethics Committee (0112-17-ZIV).

**Figure 1:**
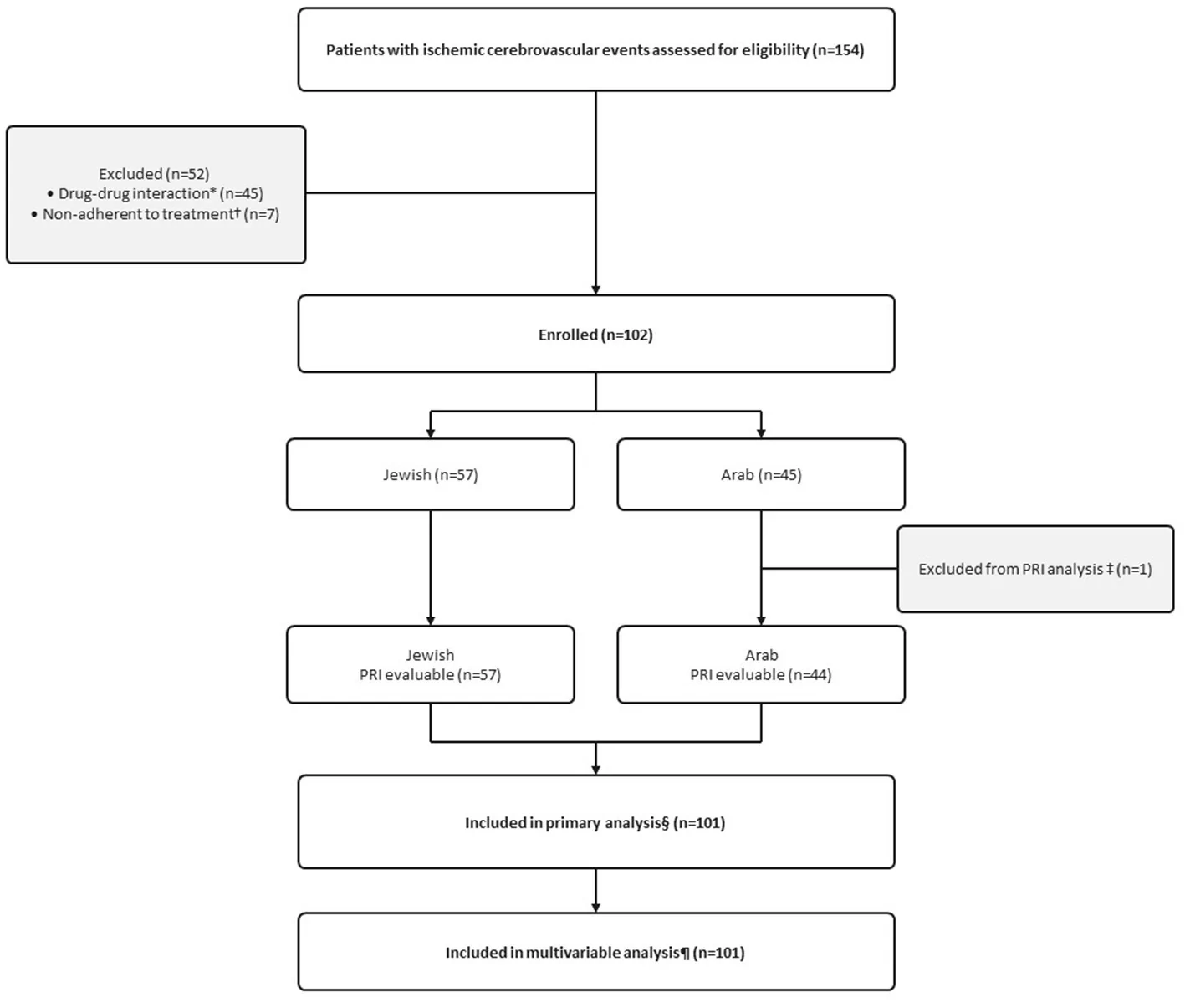
PRI, platelet reactivity index (VASP assay) ^*^ Drug-drug interation: proton-pump inhibitor, calcium-channel blocker, or warfarin use. † Non-adherence to treatment was determined by patient report ‡ One enrolled Arab patient did not have an evaluable VASP PRI measurement § Analyses involving PRI were performed in the 101 patients with an evaluable mesurement (57 Jewish, 44 Arab) ¶ Multivariable analysis population shown as identical to the primary-analysis cohort (n=101)

A study physician recruited and enrolled adult patients with ischemic stroke or transient ischemic attack (TIA) who fulfilled one of the following clopidogrel regimens for secondary prevention: (1) a loading dose of clopidogrel (300 mg in 62 patients and 600 mg in one patient) followed by at least two addition-al days of 75 mg daily maintenance (63 patients), or (2) at least 7 consecutive days of 75 mg daily maintenance dosing (39 patients). All patients received information about the study and provided written informed consent. To avoid potential interactions affecting clopidogrel metabolism, patients receiving proton pump inhibitors, calcium channel blockers, or warfarin concurrently were excluded to minimize pharmacokinetic interactions (Bonello et al., 2010). Additional exclusion criteria included as well documented nonadherence to therapy determined by patient report at the time of screening, and inability to provide informed consent.

Ethnicity was self-reported and categorized as Jewish or Arab. Demographic and clinical variables included age, sex, body mass index (BMI), prior myocardial infarction (MI), prior cerebrovascular accident (CVA) or TIA, hypertension, hyperlipidemia, diabetes mellitus, smoking status, renal dysfunction, and inflammatory conditions.

VASP platelet reactivity index (PRI) measurements were available for 101 patients; one enrolled Arab patient did not have an evaluable PRI measurement. Accordingly, analyses involving PRI were performed in 101 patients (57 Jewish and 44 Arab).

Recorded demographic and clinical variables included age, sex, ethnicity, medication history, and comorbidities such as diabetes mellitus, elevated BMI, hyperlipidemia, renal impairment, inflammatory conditions, and smoking status.

### 2.2 Blood sample collection and VASP phosphorylation assay

Blood sample collection and VASP phosphorylation assay Platelet P2Y12 receptor inhibition was quantified via the VASP assay using the PLT VASP/P2Y12 kit (BioCytex, Marseille, France) according to manufacturer instructions and recommended procedures for platelet aggregation and flow cytometry-based assays (Polack et al., 2001; Roederer, 2001; Schmitz et al., 1998).

Venipuncture was performed with a 19-gauge needle, discarding the first 2.5 mL to minimize artifactual platelet activation. Blood samples were collected into 3.2% sodium citrate tubes and gently inverted twice immediately after collection to ensure anti-coagulation. They were stored at room temperature (18–25 °C) and processed within 48 h, a timeframe validated by the manufacturer for this assay (Polack et al., 2001). Aliquots were incubated with either PGE1 alone or PGE1 plus ADP. Following cellular permeabilization, phosphorylated VASP was labeled by indirect no wash immunofluorescence using a specific monoclonal antibody. Dual-color flow cytometry analysis compared the two tested conditions to quantify ADP's capacity to inhibit VASP phosphorylation for each sample.

The PLT VASP/P2Y12 kit assesses specific platelet ADP receptor (P2Y12) antagonism by flow cytometry (Gaglia et al., 2011; Roederer, 2001; Schmitz et al., 1998). VASP is an intracellular platelet protein whose phosphorylation state is regulated by the cyclic Adenosine Monophosphate (cAMP) signaling pathway. Prostaglandin E1 (PGE1) activates this cascade and promotes VASP phosphorylation, whereas adenosine diphosphate (ADP) inhibits it through P2Y12 receptor activation. In the assay, VASP phosphorylation reflects P2Y12 inhibition; conversely, low phosphorylation indicates active P2Y12 signaling. Effective clopidogrel action is shown by sustained PGE1-induced VASP phosphorylation despite ADP exposure, demonstrating P2Y12 blockade (Gaglia et al., 2011).

The assay's reported intra-assay coefficient of variation (CV) is < 5%, and inter-assay CV is < 8% according to manufacturer validation data. Intra-patient variability was not assessed in this study and is acknowledged as a limitation.

The PRI was calculated from corrected mean fluorescence intensities (MFIc) measured in the presence of PGE1 alone or PGE1 plus ADP simultaneously: PRI = [(MFIc PGE1 – MFIc (PGE1 + ADP)) / MFIc PGE1] x 100 (BioCytex. PLT VASP/P2Y12 kit: instruction for use. Marseille, France). The results were reported as PRI percentage.

For the purposes of this exploratory study, patients were categorized according to PRI as follows: PRI < 50% was considered an adequate response; PRI 50%−69% was considered an intermediate response; and PRI ≥70% was operationally defined as high residual platelet reactivity (referred to as clopidogrel resistance in the analyses). These categories should not be interpreted as universally accepted stroke-specific definitions. In particular, HPR has also been defined using lower thresholds in other clinical settings, and the VASP-PRI thresholds used here were derived primarily from coronary populations.

### 2.3 Statistical analysis

Continuous variables were summarized as median (Me) and interquartile range (IQR), since the data were not normally distributed. Categorical data were described using frequencies (*n*) and percentages. Comparison of continuous variables between groups was performed using the Mann-Whitney non-parametric test. Relationships between categorical variables and ethnic groups were examined using the Chi-square test or Fisher's exact test depending on the sample size. Multivariable logistic regression analysis was performed to identify factors associated with operationally defined high residual platelet reactivity (PRI ≥70%). The model was based on the 101 patients with an evaluable PRI measurement, with no additional missing covariate data. Results are presented as odds ratios (ORs) with 95% confidence intervals (CIs). Results are presented as odds ratios (ORs) with 95% confidence intervals (CIs). A *p*-value < 0.05 was considered statistically significant. No formal *a priori* sample-size calculation was performed because this was an exploratory, single-center observational study. The ethnic comparison and predictor analyses were considered hypothesis-generating, and nonsignificant results were not interpreted as evidence of equivalence. No adjustment for multiple comparisons was performed because of the exploratory nature of the analyses. All statistical analyses were performed using SPSS version 31 (SPSS Inc., Chicago, IL, USA).

## 3 Results

### 3.1 Patient demographic and clinical characteristics

The study included 102 patients enrolled from November 2020 through November 2022 (Figure 1). Demographic and clinical data are summarized in Table 1. The median age was 67.0 (60–75) years, and 62.7% of patients were male. A high prevalence of cardiovascular risk factors was observed: 42.2% had diabetes mellitus, 55.9% had hypertension, and 58.8% had hyperlipidemia. Figure 2 presents the distribution of clinical risk factors according to ethnicity. The median body mass index (BMI) was 27.5 (25.5–30.5) kg/m^2^, and 17.8% of patients were current smokers. All patients received clopidogrel. The patients were divided into two ethnic groups: 57 Jewish patients (55.9%) and 45 Arab patients (44.1%). Jewish patients were significantly older than Arab patients (median age 69 vs. 64 years, *p* = 0.008). Arab patients had a significantly higher BMI than Jewish patients (28.6 vs. 26.8 kg/m^2^, *p* = 0.019). No other demographic or clinical variables differed significantly between the groups. Concomitant aspirin or other antiplatelet therapy (was recorded in 85 of 102 patients, of whom 41 (48%) were receiving concomitant antiplatelet therapy; this information was not available for the remaining 17 patients.

**Table 1:** Demographic and clinical characteristics of the study participants according to their ethnic group.

Variables	All patients (*n* = 102)	Jewish (*n* = 57)	Arab (*n* = 45)	*p*
Gender (*n*, %)				
Male	64, 62.7	37, 64.9	27, 60.0	0.610
Female	38, 37.3	20, 35.1	18, 40.0	
Age, yr. (med, IQR)	67, 60–75	69, 64–78	64, 55–72	**0.008**
BMI, kg/m^2^ (med, IQR)	27.5, 25.5–30.5	26.8, 24.5–30.4	28.6, 26.2–31.8	**0.019**
MI (*n*, %)	4, 3.9	2, 3.5	2, 4.4	0.998
CVA (*n*, %)	56, 54.9	31, 54.4	25, 55.6	0.906
TIA	27, 26.5	14, 24.6	13, 28.9	0.623
Hypertension (*n*, %)	57, 55.9	30, 52.6	27, 60.0	0.457
Hyperlipidemia (*n*, %)	60, 58.8	32, 56.1	28, 62.2	0.535
Smoking (*n*, %)	18, 17.8	10, 17.5	8, 17.8	0.975
Diabetes (*n*, %)	43, 42.2	21, 36.8	22, 48.9	0.221
Inflammation (*n*, %)	5, 4.9	2, 3.5	3, 6.7	0.652
Renal dysfunction (*n*, %)	8, 7.8	4, 7.0	4, 8.9	0.727

IQR, interquartile range; BMI, body mass index; MI, myocardial infarction; CV, cerebrovascular accident; TIA, transient ischemic attack. CVA and TIA represent prior cerebrovascular events. MI represents prior myocardial infarction. Hypertension, hyperlipidemia, diabetes, renal dysfunction, and inflammatory conditions represent baseline diagnoses at admission. Inflammation was defined as a documented inflammatory diagnosis or elevated CRP (>10 mg/L) at admission. *p*-values were derived using Chi-square or Fisher's exact test as appropriate to assess the association between categorical variables and ethnicities. Comparison of continuous variables was analyzed by the Kruskal–Wallis test. Bold values indicate statistical difference (*p* < 0.05).

**Figure 2:**
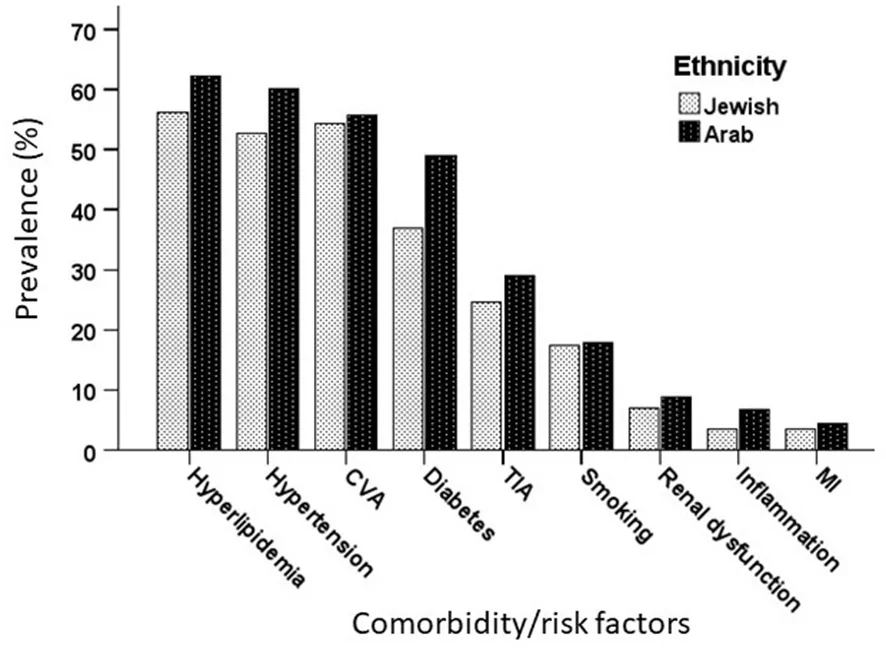
CVA, cerebrovascular accident; TIA, transient ischemic accident; MI, myocardial infarction.

### 3.2 Platelet reactivity index (PRI)

A valid VASP-PRI measurement was available for 101 patients (57 Jewish and 44 Arab); one Arab patient had no evaluable PRI measurement because of a technical failure of the VASP assay. Therefore, analyses of platelet reactivity were based on 101 patients.

The median PRI for all 101 patients was 65.5 (49.1–80.3), with no significant difference between groups: Jewish patients had a median PRI of 61.1 (17.7–77.4), whereas Arab patients had a median PRI of 68.7 (50.9–81.0) (*p* = 0251).

Patients were classified according to PRI as adequate responders (PRI < 50%), intermediate responders (PRI 50%−69%), and patients with high residual platelet reactivity (PRI ≥ 70%). Overall, 42.6% of patients had PRI ≥70%, 29.7% had PRI 50%−69%, and 27.7% had PRI < 50%. Among Jewish patients, 38.6% had PRI ≥70%, 31.6% had PRI 50%−69%, and 29.8% had PRI < 50%, compared with 47.7%, 27.3%, and 25.0%, respectively, among Arab patients. No significant differences in the distribution of response categories were observed between the two ethnic groups (*p* = 0.654).

Analysis of the continuous VASP-PRI showed no statistically significant difference between groups. The median PRI percentage was 65.5% (IQR 49.1–80.3) in the overall cohort, 61.1% (IQR 47.7–77.4) among Jewish patients, and 68.7% (IQR 50.9–81.0) among Arab patients (*p* = 0.251). Higher PRI values indicate greater residual platelet reactivity rather than greater clopidogrel inhibition.

When patients were categorized according to the operational definition of high residual platelet reactivity (PRI ≥70%), the prevalence did not differ significantly between Jewish and Arab participants. These findings indicate that no statistically significant ethnic difference in clopidogrel pharmacodynamic responsiveness was detected in this cohort; however, the study was not powered to exclude modest between-group differences, and the ethnic comparison should therefore be interpreted as exploratory.

PRI did not differ significantly between patients who received a loading dose and those treated with maintenance therapy alone (median 64.9 [IQR 42.9–79.8] vs. 66.5 [IQR 51.5–80.3]; Mann-Whitney, *p* = 0.46). Given that only one patient received a 600 mg loading dose, a separate comparison between 300 mg and 600 mg could not be meaningfully performed.

Overall, a substantial proportion of patients had elevated residual platelet reactivity according to the operational PRI threshold used in this study, with no significant differences in platelet reactivity observed between the two ethnic groups (Table 2).

**Table 2:** VASP platelet reactivity index (PRI) among patients with an evaluable measurement, according to ethnic group.

Variables	All patients (*n* = 101)	Jewish (*n* = 57)	Arab (*n* = 44)	*p*
PRI, % (med, IQR)	65.5, 49.1–80.3	61.1, 47.7–77.4	68.7, 50.9–81.0	0.251
PRI category (*n*, %)				
< 50% (adequate response)	28, 27.7	17, 29.8	11, 25.0	0.654
50%−69% (intermediate response)	30, 29.7	18, 31.6	12, 27.3	
≥70% (high residual platelet reactivity)	43, 42.6	22, 38.6	21, 47.7	
PRI category, binary (*n*, %)				
< 70%	58, 57.4	35, 61.4	23, 52.3	0.357
≥70%	43, 42.6	22, 38.6	21, 47.7	

PRI, Platelet reactivity index (VASP assay). One enrolled Arab patient (of 45) did not have an evaluable PRI measurement and is excluded from this table; denominators reflect the 101 patients with an evaluable PRI measurement.

*p*-values were derived using the Mann–Whitney *U*-test (continuous PRI).

Clinical characteristics according to platelet reactivity response are presented in Table 3. No significant differences were observed patients with PRI < 70% and those with PRI ≥70% with respect to sex (*p* = 0.464), age (*p* = 0.858), or risk factors (*p* = 0.774). In contrast, BMI was significantly higher among non-responders compared with responders/partial responders (*p* = 0.009).

**Table 3:** Clinical characteristics according to VASP platelet reactivity index (PRI).

Variables	PRI < 70% (adequate/intermediate response) (*n* = 58)	PRI ≥70% (high residual platelet reactivity) (*n* = 43)	*p*
Gender (*n*, %)			
Male	35, 60.3	29, 67.4	0.464
Female	23, 39.7	14, 32.6	
Age, years (med, IQR)	68, 62–74	66, 58–78	0.858
BMI, kg/h^2^ (med, IQR)	26.6, 24.6–29.3	29.1, 21.1–31.6	**0.009**
Risk factors (med, IQR)	3, 1–4	3, 2–4	0.774

PRI-Platelet reactivity index (VASP assay). Based on the 101 patients with an evaluable PRI measurement.

*p*-values were derived using the Mann–Whitney *U*-test (continuous variables) or the Chi-square/Fisher's exact test (categorical variables), as appropriate. Bold values indicate statistical difference (*p* < 0.05).

Multivariable logistic regression analysis was performed to evaluate factors associated with operationally defined high residual platelet reactivity (PRI ≥70%; *n* = 101; Table 4 and Figure 3); the covariates entered into the model are listed in Table 4. Male sex was independently associated with a lower likelihood of high residual platelet reactivity (OR = 0.28, 95% CI: 0.08–0.93, *p* = 0.038). Similarly, smoking was associated with reduced odds of high residual platelet reactivity (OR = 0.18, 95% CI: 0.04–0.81, *p* = 0.026). In contrast, age (OR = 1.00, 95% CI: 0.94–1.05, *p* = 0.872), BMI (OR = 1.04, 95% CI: 0.92–1.19, *p* = 0.531), Jewish ethnicity (OR = 0.83, 95% CI: 0.24–2.91, *p* = 0.770), hyperlipidemia (OR = 2.34, 95% CI: 0.49–11.14, *p* = 0.287), and diabetes mellitus (OR = 0.69, 95% CI: 0.14–3.46, *p* = 0.649) were not significantly associated with high residual platelet reactivity.

**Table 4:** Multivariable logistic regression model for factors associated with high residual platelet reactivity (PRI ≥70%).

Variables	Values	OR	95% CI	*p*
Age	continues	1.00	0.94–1.05	0.872
BMI	continues	1.04	0.92–1.19	0.531
Gender	1 = male	0.28	0.08–0.93	**0.038**
Ethnicity	1= Jewish	0.83	0.24–2.91	0.770
Hyperlipidemia	1 = yes	2.34	0.49–11.14	0.287
Smoking	1 = yes	0.18	0.04–0.81	**0.026**
Diabetes	1 = yes	0.69	0.14–3.46	0.649

PRI- platelet reactivity index (VASP assay), OR-Odd ratio, CI-Confidence interval.

Model based on the *n* = 101 patients with an evaluable PRI measurement, no additional patients were excluded for missing covariate data. Bold values indicate statistical difference (*p* < 0.05).

**Figure 3:**
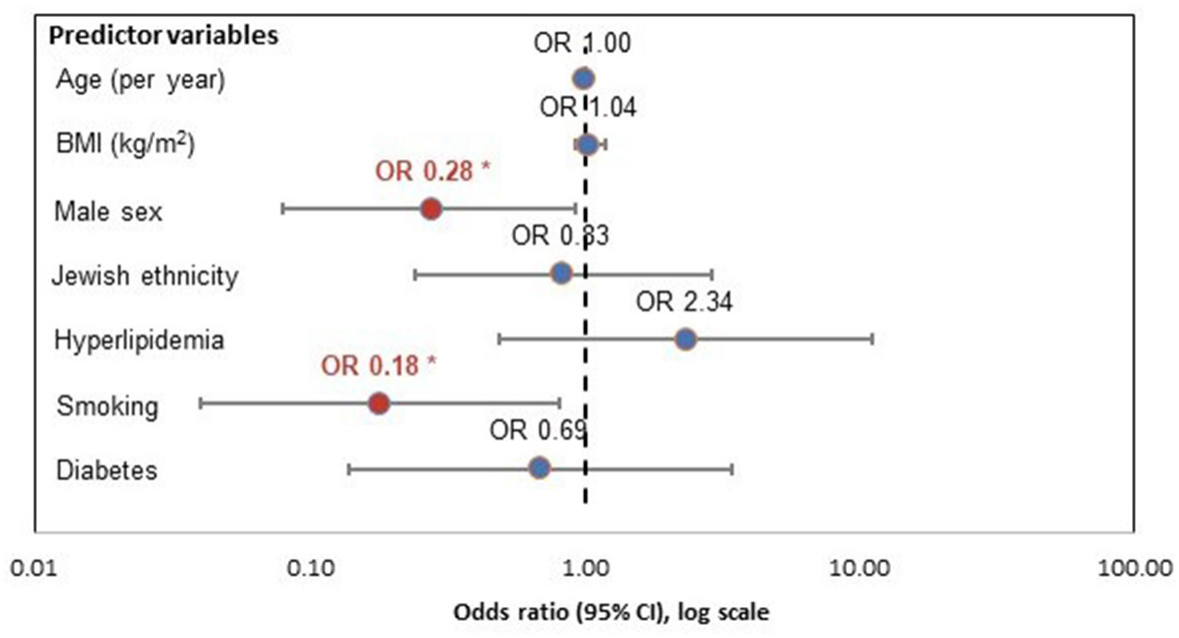
BMI, body mass index; OR, odds ratios

## 4 Discussion

In this prospective cohort of patients with ischemic cerebrovascular events in Northern Israel, no statistically significant difference in VASP-PRI was detected between Jewish and Arab patients. This finding is consistent with prior reports describing overlapping ranges of clopidogrel responsiveness in both populations studied separately (Hulot and Fuster, 2009; Levine et al., 2014; Mega et al., 2009; Schmitz et al., 1998). However, given the limited sample size (57 Jewish and 45 Arab patients), this study was underpowered to exclude a modest true difference in platelet reactivity between groups. The ethnic comparison should therefore be interpreted as exploratory and hypothesis-generating rather than as evidence that ethnic background has no influence on clopidogrel responsiveness. Biological resistance to clopidogrel is influenced by diverse factors, including CYP polymorphisms, especially loss-of-function CYP2C19 alleles, as well as clinical and demographic variables such as age, renal function, diabetes, and body mass index (BMI) (Brandt et al., 2007; Buonamici et al., 2007; Geisler et al., 2008; Hochholzer et al., 2010, 2011; Hulot et al., 2006; Matetzky et al., 2004; Mega et al., 2009; Shuldiner et al., 2009; Trenk et al., 2008). The prevalence of CYP2C19 loss-of-function alleles varies markedly by ethnicity (up to 65% in East Asian vs. approximately 30% in Caucasians) (Levine et al., 2014). We hypothesized that genetic differences and varying cardiovascular risk profiles across ethnic groups might translate into differential clopidogrel responsiveness. Although Arab patients in our cohort tended to have higher rates of certain cardiovascular risk factors, only hyperlipidemia reached statistical significance. Despite this, no ethnic differences in clopidogrel response were detected. This may suggest that ethnicity alone is not the dominant determinant of clopidogrel response in the Northern Israeli setting, or that genetic and environmental contributors to metabolism are more similar between these groups than expected.

A major limitation of our study is the absence of CYP2C19 genotyping. CYP2C19 loss-of-function alleles (^*^2, ^*^3) are well-established determinants of reduced clopidogrel active metabolite formation and increased platelet reactivity (Hulot et al., 2006; Mega et al., 2009; Shuldiner et al., 2009). Pharmacogenetic studies have demonstrated variability in allele frequencies across ethnic groups, including among Jewish subpopulations (Ashkenazi, Sephardi, and others) and Arab populations in the Middle East (Ali and Elewa, 2019; Ali et al., 2022; Alkattan et al., 2022; Yang et al., 2014). Without genotyping data, we cannot determine whether the observed variability in platelet responsiveness in our cohort reflects underlying genetic polymorphism distribution, clinical factors, or their interaction. Future investigations should incorporate pharmacogenomic testing to better delineate the relative contributions of genetic and clinical determinants of clopidogrel response (Lee et al., 2022; Scott et al., 2013, 2011).

The novelty of this study lies in the direct head-to-head comparison within a single geographic region and healthcare system, minimizing system-level confounding. A substantial proportion of patients demonstrated elevated residual platelet reactivity according to the operational threshold used in this study: 43% had PRI ≥70%, 30% had PRI 50%−69%, and 28% had PRI < 50%; thus, 72% had PRI ≥50%. However, this distribution should be interpreted cautiously because VASO-PRI thresholds for HPR have primarily been established in coronary populations, and no universally accepted stroke/TIA-specific threshold has been validated. Previous studies in patients with ischemic stroke or TIA have reported substantial variability in the prevalence of HPR depending on the platelet-function assay and cutoff used; a systematic review and meta-analysis of 21 studies including 4,312 patients reported a pooled prevalence of approximately 28% (Alakbarzade et al., 2020). Importantly, although previous studies and meta-analyses have reported associations between HPR and recurrent ischemic events or poorer clinical outcomes (Alakbarzade et al., 2020; Fiolaki et al., 2017; Krishna et al., 2012; Matetzky et al., 2004; Wiśniewski et al., 2020; Yi et al., 2018), our study was designed as a pharmacodynamic observational study and did not assess recurrent stroke, other vascular events, functional outcomes, or bleeding. Therefore, the present findings cannot establish the prognostic significance of the observed PRI values or demonstrate that patients with elevated PRI experienced worse clinical outcomes. Several factors may contribute to variability in clopidogrel responsiveness, including the acute inflammatory and prothrombotic state associated with ischemic cerebrovascular events, comorbid conditions such as diabetes mellitus, treatment exposure, and genetic factors affecting clopidogrel metabolism (Fiolaki et al., 2017; Hulot et al., 2006; Mega et al., 2009; Scott et al., 2012, 2011; Shuldiner et al., 2009). However, these potential mechanisms were not directly investigated in the present study and therefore cannot be established as explanations for the observed distribution of PRI values.

Current evidence does not support routine platelet-function testing as an established strategy for improving clinical outcomes in patients with ischemic stroke or transient ischemic attack. The 2021 AHA/ASA guideline for secondary stroke prevention does not recommend routine platelet-function testing to guide antiplatelet therapy, reflecting the limited evidence that test-guided treatment modification improves clinical outcomes (Kleindorfer et al., 2021). More recently, the 2026 AHA/ASA guideline for the early management of acute ischemic stroke identified the reliability and clinical utility of clopidogrel loss-of-function testing, as well as whether changing or increasing antiplatelet therapy based on such testing improves outcomes, as areas requiring further investigation (Prabhakaran et al., 2026). Accordingly, our findings should not be interpreted as supporting routine platelet-function testing or treatment modification based on VASP-PRI. In particular, the present study does not establish that increasing the clopidogrel dose, switching antiplatelet therapy, or adding antiplatelet agents according to PRI improves clinical outcomes in patients with ischemic stroke or TIA. Although treatment strategies aimed at overcoming HPR have been investigated predominantly in coronary populations, these findings cannot be directly extrapolated to cerebrovascular populations because of differences in disease characteristics, treatment goals, and bleeding risk. In particular, prasugrel should not be considered an alternative treatment in patients with a history of stroke or TIA, as prior stroke or TIA is a contraindication to prasugrel therapy (Levine et al., 2016). Furthermore, long-term dual antiplatelet therapy is generally not recommended for secondary stroke prevention because of the increased risk of bleeding, while short-term dual antiplatelet therapy is reserved for selected clinical scenarios (Kleindorfer et al., 2021; Prabhakaran et al., 2026). Prospective, adequately powered outcome-based studies are therefore needed to determine whether platelet-function- or genotype-guided antiplatelet strategies can improve clinical outcomes while maintaining an acceptable bleeding risk in patients with ischemic cerebrovascular disease.

Strengths of this study include its prospective design and the use of VASP phosphorylation as a specific measure of P2Y12 receptor inhibition, which is considered a reliable and specific surrogate marker for clopidogrel responsiveness. Other platelet function tests include light transmission aggregometry (LTA), which assesses both P2Y1 and P2Y12 pathways, and the VerifyNow P2Y12 assay, which provides a practical point-of-care measure. Despite multiple available assays, there is currently no consensus on a standard methodology or a universally accepted definition of clopidogrel resistance (Bonello et al., 2010; Mallouk et al., 2012). Different assays and cutoffs may classify the same patient differently depending on the specific biological pathway assessed (Bonello et al., 2010; Gaglia et al., 2011; Mallouk et al., 2012). This lack of standardization represents a significant barrier to incorporating platelet function testing into routine clinical practice.

In this context, it is important to note that the PRI cutoff values used in this study were originally validated primarily in coronary artery disease and percutaneous coronary intervention populations (Bonello et al., 2010; Tantry et al., 2013). Currently, no stroke-specific consensus thresholds for VASP-derived high on-treatment platelet reactivity exist. As a result, most ischemic stroke studies adopt coronary-derived cutoffs to ensure comparability across studies (Fiolaki et al., 2017; Zhou et al., 2022). However, the pathophysiological and thrombotic milieu in acute ischemic stroke may differ from coronary disease, potentially influencing optimal threshold definition. Therefore, extrapolation of these cutoffs to stroke populations should be interpreted cautiously, and future studies should aim to validate stroke-specific platelet reactivity thresholds linked to clinical outcomes (Krishna et al., 2012; Yi et al., 2018; Zhou et al., 2022). Establishing standardized assays and unified cutoff values is essential to optimize and individualize antiplatelet therapy (Bonello et al., 2010; Tantry et al., 2013).

The inclusion of patients from a single medical center in Northern Israel may limit the generalizability of our findings to other geographic regions or healthcare contexts. The specific ethnic composition of our study population (Jewish and Arab populations from the Galilee region) may not be representative of other multiethnic cohorts. Importantly, with 57 Jewish and 45 Arab patients, the study was statistically underpowered to detect anything other than relatively large difference in platelet reactivity between ethnic groups; the negative comparison should not be interpreted as definitive evidence of equivalence, and the ethnic subgroup analysis is best regarded as exploratory and hypothesis-generating, warranting confirmation in larger, adequately powered cohorts. The exact interval between clopidogrel dosing and blood sampling was not systematically recorded in this cohort and could not be reliably reconstructed retrospectively, precluding a more granular pharmacodynamic analysis of dosing timing. As noted above, the absence of CYP2C19 and broader pharmacogenomic testing represents a central limitation, precluding mechanistic interpretation of the ethnic comparisons and response variability observed. Future studies should incorporate genotyping to delineate the relative contributions of genetic vs. clinical determinants of clopidogrel resistance across ethnic groups (Hulot et al., 2006; Lee et al., 2022; Mega et al., 2009; Scott et al., 2013, 2011; Shuldiner et al., 2009).

## 5 Conclusion

This prospective study demonstrates a high prevalence of elevated residual platelet reactivity among patients with ischemic cerebrovascular events in Northern Israel, with no statistically significant differences in VASP-PRI between Arab and Jewish patients. However, given the limited statistical power, ethnic comparisons should be considered exploratory and hypothesis-generating rather than definitive. The absence of CYP2C19 genotyping and longitudinal clinical outcomes further limits mechanistic and clinical interpretation. These findings do not establish that platelet-function testing or treatment modification according to PRI improves outcomes. Larger, adequately powered multicenter prospective studies incorporating pharmacogenomic assessment, standardized definitions of platelet reactivity, and longitudinal clinical outcome follow-up are warranted to clarify the clinical relevance of ethnic and genetic determinants of clopidogrel responsiveness in ischemic cerebrovascular populations.

## Data Availability

The raw data supporting the conclusions of this article will be made available by the authors, without undue reservation.
